# Comparative proteomic analysis between tumor tissues and intratumoral exosomes from lung adenocarcinoma patients identifies PAFAH1B3 as an exosomal protein key for initiating metastasis in lung adenocarcinoma

**DOI:** 10.1016/j.heliyon.2024.e39859

**Published:** 2024-10-28

**Authors:** Congcong Wang, Ling Xiao, Ling Gao, Jia Wu, Siliang Wang, Miao-Miao Zheng, Chen-Tai Qin, Xian-ge Huang, Lei Zhou, Wei-jie Xu, He-gen Li, Wen-Lian Chen, Li-hua Zhu, Xing Jin

**Affiliations:** a Cancer Institute, Longhua Hospital, Shanghai University of Traditional Chinese Medicine, Shanghai, 200032, China; b Shanghai Frontiers Science Center of Disease and Syndrome Biology of Inflammatory Cancer Transformation, Shanghai, 200032, China; c Department of Oncology, Longhua Hospital, Shanghai University of Traditional Chinese Medicine, Shanghai, 200032, China

**Keywords:** Proteomics, Exosome, PAFAH1B3, Lung adenocarcinoma, Migration

## Abstract

Mounting evidence strongly indicates that exosomes are pivotal in the advancement of cancer, yet the overarching profile of exosomal proteins and their contribution to lung adenocarcinoma (LUAD) progression remain underexplored. In our investigation, we isolated exosomes from treatment-naive LUAD (n = 20) and paired normal adjacent tissues (NATs), and conducted integrated proteomic on the acquired exosomes and source tissues to ascertain origin characteristics and potential therapeutic targets of the exosomal proteins in LUAD. The omics data revealed the overall landscape of exosomal proteins from tissues in LUAD, underscoring the profound linkage between exosomal proteins and tumor metastasis. Integrated analysis indicated a significant overlap in protein species, demonstrating high concordance between exosomal proteins and those in their originating tissues. However, only a small subset showed significant positive correlation in protein abundance between exosomes and their source tissues. Notably, we pinpointed five proteins (DDX18, DNAJA3, PAFAH1B3, BAG6, and CAD). Significantly, platelet activating factor acetylhydrolase 1b catalytic subunit 3 (PAFAH1B3), an essential serine hydrolase within cellular metabolic processes, stood out as the singular protein closely associated with disease-free survival (DFS) of patients. Cell invasion and migration assays further substantiated that PAFAH1B3 promoted metastasis of LUAD via the exosomal release pathway. Furthermore, analysis of public databases validated elevated *PAFAH1B3* expression in LUAD and linked it to poor patient survival outcomes. Overall, our research positioned PAFAH1B3 as a promising candidate for prognostic marker and potential therapeutic target in lung cancer treatment.

## Graphical abstract

**Figure:**
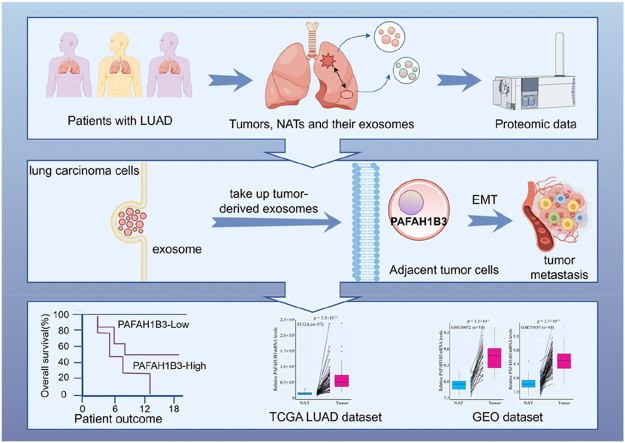

## 1 Introduction

Lung cancer stands as one of the most fatal malignancies, holding the second-highest incidence rate and the highest mortality worldwide. Its prognosis remains bleak, with a median overall survival (OS) spanning from 6.0 to 12.7 months, and a 5-year OS rate seldom surpassing 25 % [1,2]. Histopathologically, lung cancer is primarily divided into small cell lung cancer (SCLC, approximately 15 %) and non-small cell lung cancer (NSCLC, approximately 85 %) [3,4]. Within NSCLC, Lung adenocarcinoma (LUAD) emerges as the foremost subtype, responsible for around 78 % of cases [3]. Despite remarkable advances in clinical interventions, such as surgery, radiotherapy, chemotherapy, targeted therapy, and immunotherapy, metastasis still occurs in roughly 30%–55 % of patients [5,6]. Therefore, it is critical to identify key targets driving metastasis and develop effective therapeutic strategies, with the ultimate aim of improving the prognosis for patients with LUAD.

Exosomes, cellularly-secreted extracellular vesicles ranging from 40 to 160 nm in diameter [7], have gained considerable attention in recent years. Accumulating evidence has established their pivotal role in reshaping the tumor microenvironment and propelling tumor metastasis through their function as carriers of bioactive molecules, including proteins, mRNA, DNA, microRNA, lipids etc. [8,9]. At the forefront of exosomal protein research, the focus has increasingly shifted toward identifying specific molecular targets. Exosomes derived from lung cancer cells have been found to possess the unique capability to alter the phenotype of macrophages within the pre-metastatic niche via TLR2 and NLRP6 signaling pathways, thus significantly contributing to tumor progression [10,11]. Notably, PFN2, an exosome protein from lung cancer cells, has been shown to markedly enhance migration and tube formation in vascular endothelial cells [12]. Additionally, fibroblasts in the tumor microenvironment are able to transfer the Snail 1 protein to receiving cancer cells through exosomes, which induced epithelial-mesenchymal transition (EMT) in lung cancer cells and promoted tumor angiogenesis [13]. These findings underline the significance of exosomal proteins as crucial mediators of intercellular crosstalk and communication. However, there is still a lack of in-depth understanding of comprehensive characteristics of exosomes in lung cancer, with the majority of studies focusing solely on comparative analyses of liquid biopsy [[14], [15], [16], [17]].

In this investigation, we utilized proteomic methodologies to execute an exhaustive omics dissection of 20 LUAD tumors, along with their normal adjacent tissues (NATs) and the exosomes they release. Our dataset provided valuable insights into the relationship between tissue proteins and exosomal proteins. Through comprehensive subsequent proteomic analysis and in vitro experiments, we have delineated the role of the exosomal protein PAFAH1B3 in augmenting the metastatic propensity of lung cancer cells. Significantly, publicly accessible dataset revealed that PAFAH1B3 is not only aberrantly expressed but also harbors potential as a robust prognostic biomarker for lung cancer prognosis and therapeutics.

## 2 Materials and methods

### 2.1 Clinical specimen collection and ethical approval

We defined stringent criteria for enrolling patients with LUAD at the Affiliated Tumor Hospital of Nantong University (ATHNU): patients with LUAD with a definitive histopathological diagnosis, no previous history of malignancy, no prior treatment, undergoing pulmonary lobectomy, and providing written informed consent in accordance with the regulations of the relevant Hospital Organizational Review Board and the Declaration of Helsinki. Paired tumors and NATs were collected for both tissue proteomic and tissue exosomal proteomic analyses using Data Independent Acquisition (DIA).

### 2.2 Proteomic analysis

Proteomic examinations were executed on the entirety of proteins extracted from the tumor tissues and NATs, alongside proteins released from their respective exosomes. Our analytic approach unfolded as follows. We initiated the process with data-dependent analysis (DDA) to build a comprehensive DDA spectrum library. The peptides were re-suspended in 0.1 % formic acid and chromatographically resolved using a C18 column (50 mm × 15 cm, 1.7 μm). The elution employed a duo of solvent systems: solvent A (a mixture of formic acid: H2O = 1:1000 [v/v]) and solvent B (a mixture of formic acid: acetonitrile = 1:800 [v/v]). Elution was performed at a flow rate of 200 nL/min. The elution gradient consisted of the following steps: 5%–8% solvent B (0–3 min); 8%–44 % solvent B (3–100 min); 44%–60 % solvent B (100–103 min); 60%–100 % solvent B (103–105 min); 100 % solvent B (105–120 min). The fractions were determined using Nano-liquid chromatography coupled with Orbitrap Exploris480 + FAIMS mass spectrometer. The raw data obtained from mass spectrometry was subjected to analysis using Proteome Discovery software in order to generate a comprehensive spectrum library.

Data Independent Analysis (DIA) mirrored the same analytical methodologies as DDA. DIA mass spectrometry data were fed into Spectronaut 14.0, leveraging the previously built spectrum library in the data processing workflow. All proteins and peptides underwent qualitative assessment with a stringent Q value cutoff of 0.01 (equivalent to FDR of 1 %). Quantitative analysis capitalized on the peak areas observed at the MS2 stage, wherein peptide abundances were deduced by summing the peak areas of their specific MS2 fragment ions. Concomitantly, protein abundances were deduced by summing the abundances of their constituent peptides.

### 2.3 Proteomic data analysis

#### 2.3.1 Missing value imputation

Our tissue proteome exploration yielded an inventory of 9,030 distinct proteins across 40 samples. Similarly, proteomic analysis of exosomes surfaced 7,046 unique proteins within 40 exosomal samples. We adopted an exclusion criterion that filtered out proteins demonstrating more than a 50 % missing value rate within LUAD and NAT samples. For the remainder, missing values were imputed using the smallest non-missing value available within the dataset [18].

#### 2.3.2 Differential expression examination

For proteomic data, we performed the Shapiro-Wilk normality test to assess the distribution characteristics of the data. In the tissue proteomic dataset, 59.11 % of proteins in the clinical tumor tissues and 40.13 % of proteins in matched NATs displayed skewed distribution (Table S1). Similarly, in the tissue-derived exosomal proteomic dataset, 60.89 % of proteins in the LUAD-derived exosomes and 56.29 % of exosomal proteins from paired NATs, both displaying skewed distribution patterns (Table S2). Subsequently, we calculated P-values using the nonparametric and paired two-class Wilcoxon rank-sum tests, refining these further with a Bonferroni adjustment. We employed the R package qvalue (v3.18) (http://github.com/jdstorey/qvalue) to compute FDR q-values. Variable important in projection (VIP) scores were ascertained using the ropls package (v3.18) (https://github.com/SamGG/ropls). We computed Fold Change (FC) values by dividing the median expression levels of each protein in LUAD tumors by the median levels observed in NATs. Proteins deemed as differentially expressed between LUAD and NATs were selected upon meeting the Bonferroni-adjusted *p* < 0.05, FDR q < 0.05, VIP >1, and FC cutoff value of 2.

### 2.4 Cell culture

The human LUAD cell lines A549 and NCI-H1299, both sourced from ATCC (Manassas, VA, USA), were propagated in Dulbecco's Modified Eagle Medium (DMEM, cat#L110KJ, Thermo Scientific, Waltham, MA, USA). This medium was enriched with 10 % fetal bovine serum (FBS, cat#10099141C, Thermo Scientific, Waltham, MA, USA) and a cocktail of antibiotics, comprising 100 U/ml penicillin and 100 μg/mL streptomycin (cat#15140122, Thermo Scientific, Waltham, MA, USA). Cell cultures were incubated at 37 °C in a humidified environment with a 5 % CO_2_ atmosphere, ensuring optimal growth conditions.

### 2.5 Isolation and purification of exosomes

For the isolation and subsequent purification of exosomes, we utilized the differential centrifugation protocol referenced in the literature [19]. Our procedure began with cells cultivated in complete media, which were then rinsed thrice using phosphate-buffered saline (PBS). This was followed by a 48 h incubation in media supplemented with 10 % exosome-depleted fetal bovine serum (cat#C3801-0050, Biological Industries, Kibbutz Beit Haemek, Israel) to ensure minimal contamination. After this incubation period, we collected the cell culture supernatant and subjected it to an initial centrifugation at 3,000×*g* for 10 min at 4 °C, a step designed to discard cellular debris. A subsequent centrifugation at 10,000×*g* for an hour at 4 °C was employed to clear the sample of larger macromolecular complexes. The resulting supernatant underwent filtration through a 0.22 μm pore-size filter (cat#SLGPR33RB, Merck Millipore, Billerica, MA, USA) to further purify the sample by removing non-vesicular components. The filtrate was then subjected to ultracentrifugation at 120,000×*g* for 90 min at 4 °C. This high-speed spin was key for sedimenting vesicles less than 100 nm in diameter as well as co-precipitating protein aggregates. The initial pellet was resuspended in PBS and ultracentrifuged again under identical conditions to eradicate any residual contaminating proteins. The final pellet, primarily consisting of purified exosomes, was carefully resuspended in chilled PBS, ready for downstream analysis and experiments.

### 2.6 Transmission electron microscope (TEM)

To visualized the morphology and structure of exosomes, we prepared samples for TEM. Purified exosomes were immobilized on the copper grid of TEM. After staining with 3 % uranyl acetate, the grid was air-dried at room temperature and examined at magnifications of 10,000 and 40,000 using HITACHI HT7800 TEM (Hitachi, Tokyo, Japan).

### 2.7 Nanoparticle tracking analysis (NTA)

To accurately determine the size distribution and concentration of the exosomes, we employed NTA utilizing the ZetaView PMX 110 system (Particle Metrix, Meerbusch, Germany).

### 2.8 Western blot analysis

The cell and exosome samples were lysed with RIPA buffer (cat#V900854, Sigma-Aldrich, St. Louis, MO, USA) containing 1 % protease inhibitor cocktail (v/v; cat#539131, Sigma-Aldrich, St. Louis, MO, USA) and quantified using a BCA protein assay kit (cat#A53226, Thermo Scientific, Waltham, MA, USA). Subsequently, 30 μg of denatured protein (per sample) was separated by SDS-PAGE and transferred to a PVDF membranes (cat#ISEQ00010, Merck Millipore, Billerica, MA, USA). The membranes were then blocked with 5 % skimmed milk for 1 h at room temperature. Following that, the membrane was incubated overnight at 4 °C with primary antibodies, followed by incubation with secondary antibodies conjugated with Rabbit IgG-HRP (cat#7074S, Cell Signaling Technology, Boston, MA, USA) or Mouse IgG-HRP (cat#7076S, Cell Signaling Technology, Boston, MA, USA) for 1 h. The chemiluminescence signal was detected using an enhanced chemiluminescence mixture and images were visualized using a gel imaging system (Bio-Rad, Hercules, CA, USA). Primary antibodies against CD63 (cat#ab134045, Abcam, Cambridge, UK), CD81 (cat#ab109201, Abcam, Cambridge, UK), CD9 (cat#13174, Cell Signaling Technology, Boston, MA, USA), Calnexin (cat #2679, Cell Signaling Technology, Boston, MA, USA), E-cadherin (cat#3195, Cell Signaling Technology, Boston, MA, USA), N-cadherin (cat#14215, Cell Signaling Technology, Boston, MA, USA), Fibronectin (cat#26836, Cell Signaling Technology, Boston, MA, USA), Vimentin (cat#5741, Cell Signaling Technology, Boston, MA, USA), β-catenin (cat#9562, Cell Signaling Technology, Boston, MA, USA), PAFAH1B3 (cat#ab241288, Abcam, Cambridge, UK), and Actin (cat#23660-1-AP, Proteintech, Wuhan, China) were enrolled in this study.

### 2.9 Cell migration and invasion assays

The LUAD cells were suspended in 200 μL of FBS-free DMEM medium (with or without 10^9^ particles/mL of exosomes or 10 μM of GW4869) or cell culture supernatant and inoculated onto an 8 μm pore size insert (cat#3422, Corning, NY, USA) coated with 1.2 mg/mL of matrix gel (cat#354234, Corning, NY, USA). A chemoattractant, consisting of 600 μL of DMEM medium containing 20 % exosome-depleted FBS, was added to the lower chamber. After incubation for 24 h, the cells on the upper surface of the insert were gently removed using cotton swabs, while those that invaded the lower surface were fixed with 4 % paraformaldehyde (cat#BL539A, BioSharp, Hefei, China) for 30 min and stained with 0.1 % crystal violet (w/v; cat#C0775, Sigma-Aldrich, St. Louis, MO, USA). At least five images were captured for each insert using a microscope equipped with a 10× objective lens, and Image J software was employed to quantify the number of invading cells. Cell migration assays were conducted using uncoated inserts as described above.

### 2.10 CRISPR-Cas9

In our investigation, the LUAD cell lines A549 and H1299 were selected for CRISPR-Cas9 experiment as per previously established protocols [20,19]. In brief, guide RNA (gRNA) duplexes were cloned into the lenti-CRISPR-v2-puro vector, which was pre-linearized using the BsmBI endonuclease. To produce the lentiviruses, HEK293T cells were co-transfected with the lenti-CRISPR-v2-puro vector containing either non-targeting control (NC) gRNA or gRNA targeting the gene of interest, along with psPAX2 and pMD2.G packaging plasmids. This transfection was facilitated by Lipofectamine 2000 (cat#11668019, Thermo Scientific, Waltham, MA, USA), following the manufacturer's instructions. Afterward, the lentivirus-rich supernatant was collected, passed through a 0.45 μm filter (cat#SLHAR33SB, Merck Millipore, Billerica, MA, USA) to exclude any cellular contaminants, and used to infect LUAD cells. This infection process was aided by the addition of polybrene at a concentration of 8 μg/mL (cat#28728-55-4, Santa Cruz, CA, USA). Following transduction, cells were subjected to a puromycin selection regimen for 24 h to enrich for successfully transfected populations. Within this study, the following gRNAs were utilized to induce targeted gene disruptions:

gRNA of NC: 5′-AAGAAGAATTGGGGATGATG-3’;

gRNA#1 for PAFAH1B3: 5′-CCCGCAGCACCATCGGTTCG-3’;

gRNA#2 for PAFAH1B3: 5′-GAACCCGAAGTCGTCTTCAT-3’.

## 3 Results

### 3.1 Proteomic landscape of tissues and their secreted exosomes in LUAD

**Fig. 1:**
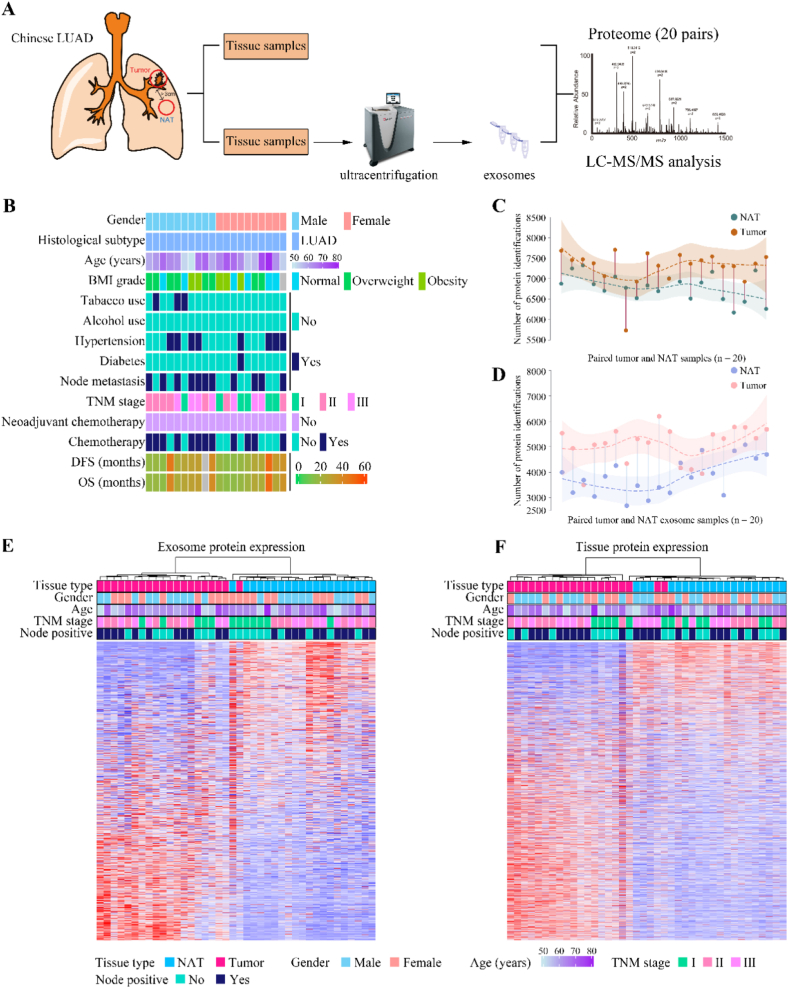
Proteomic landscape of tissues and their secreted exosomes in LUAD. (A) Overview of the experimental design using paired tumor tissues and NATs from patients with LUAD (n = 20). (B) Heatmap illustrated the demographic and histological characteristics of patients with LUAD in this study. (C and D) Identification of tissue proteins (C) and tissue-derived exosome proteins (D) in LUAD. The dashed curves, fitted using lasso regression, illustrated the distribution of protein identification in tumors (n = 20) and NATs (n = 20). The shading beneath the lasso curves represented the 95 % confidence intervals. (E and F) Unsupervised hierarchical clustering of exosome proteome (E) and tissue proteome (F) data from patients with LUAD.

We gathered 20 primary LUAD samples along with paired NATs from Chinese patients who had not received prior treatment. The experimental workflow was depicted in Fig. 1A. Rigorous quality control protocols were enforced for each specimen. LUAD regions exhibiting greater than 80 % tumor purity and NAT sections situated more than 3 cm from the tumor site were selected for analysis. Excess fat, connective, necrotic tissues, and other non-essential tissues were excised. Each tissue sample was bifurcated, one half dedicated to exosome isolation via ultracentrifugation. Detailed characterization of the exosomes, covering size, morphology, and surface biomarkers, was presented in Figs. S1 and S6. Both the exosome and the corresponding tissue fractions were subjected to proteomic scrutiny using a data-independent acquisition (DIA) methodology. Clinical characteristics of patients with LUAD were encapsulated in Fig. 1B and TableS3.

For analytical rigor in proteomics data, a Spearman's correlation coefficient was calculated across quality control (QC) runs employing HEK-293T cell samples (Fig. S2A). The average correlation coefficient among QC samples was 0.68, the variability of QC samples, data points for Full peak width at half maximum (FWHM), peak capability, and internal calibration standards indicated consistent stability of the MS platform (Figs. S2B–2E). Correlation coefficients for the 20 tumor tissue specimens ranged from 0.46 to 0.7 (mean = 0.61), while the correlations of the 20 tumor-derived exosome samples were between 0.38 and 0.72 (mean = 0.54) (Fig. S3). Proteomic analysis at the protein and peptide strata with a 1 % False discovery rate (FDR) unveiled 9030 proteins, with 8998 and 8662 proteins detected in tumors and NATs, respectively (Fig. 1C). On average, 7040 proteins were identified per sample of tissue proteomics, fluctuating from a minimum of 5728 to a maximum of 7705 for both tumor samples (Fig. 1C). The proteomic assessment also identified 7013 proteins in tumor-derived exosomes and 6305 in NAT-derived exosomes (Fig. 1D). On average, 4469 proteins were detected per proteomics sample, ranging from 2682 in NAT-derived exosomes to 6195 in tumor-derived exosomes (Fig. 1D). After discarding samples with a missing rate exceeding 50 %, unsupervised hierarchical clustering analysis exposed distinct proteomic fingerprints of tumors and those tumors-secreted exosomes compared with NATs, respectively (Fig. 1E–F). In sum, our investigation offers the inaugural comprehensive proteomic mapping of both tissue samples and tissue-derived exosomes from Chinese patients with LUAD.

### 3.2 Proteomics features of tissues and their secreted exosomes in tumors compared with NATs

**Fig. 2:**
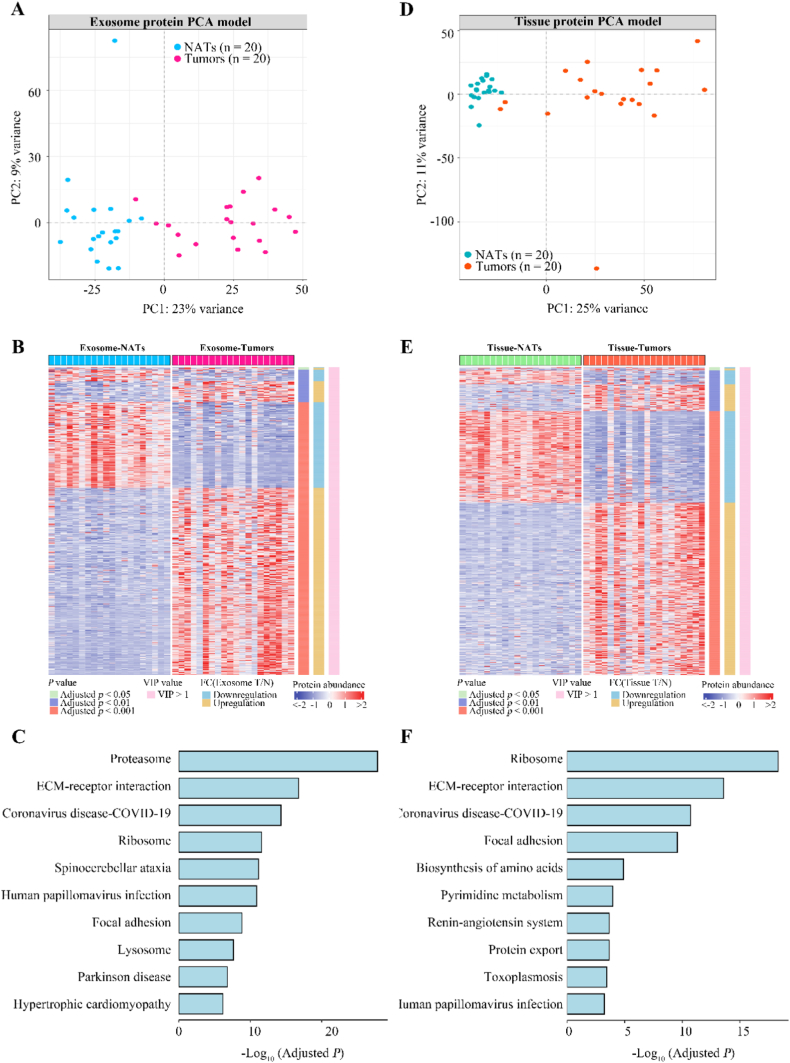
Proteomics features of tissues and their secreted exosomes in tumors Compared with NATs. (A) Principal component analysis (PCA) revealed distinctive proteomic differences between exosomes released from tumors and NATs. (B) Heatmap highlighted a total of 965 exosome proteins that were differentially expressed in tumors compared to NATs. (C) Gene Set Enrichment Analysis (GSEA) focused on differentially expressed proteins in exosome proteomics. (D) PCA was performed on the protein profiles of 20 paired tumors and NATs from patients with LUAD. (E) Identification and presentation of tissue proteins with differential expression between tumors and NATs. (F) GSEA employed to explore the biological pathways impacted by differentially expressed tissue proteins between tumors and NATs.

In the tissue-derived exosomal proteomic analysis, we identified a total of 2,910 proteins in over 50 % patients. Principal component analysis (PCA) exposed significant proteomic alterations in exosomes from tumor tissue as compared to those from NATs, thereby highlighting proteomic signatures unique to LUAD-derived exosomes (Fig. 2A). These results align with recent advances in exosome-focused liquid biopsy techniques for early-stage lung cancer detection [15,16,21]. Differential expression analysis revealed 965 exosomal proteins meeting significance thresholds (Bonferroni-adjusted *p* < 0.05, q < 0.05, VIP >1, FC cutoff of 2), with 659 proteins being upregulated and 306 downregulated in tumor-derived samples relative to NATs (Fig. 2B). Gene Set Enrichment Analysis (GSEA) demonstrated a substantial link between exosomal protein expression patterns and biological processes, including proteasome activity, extracellular matrix (ECM)-receptor interactions, ribosome functionality, focal adhesions, and various signaling cascades (Fig. 2C).

Moving to tissue proteomics, we encountered 5,911 proteins across more than half the patient cohort. PCA demarcated a clear proteome distinction between tumor and NAT samples, reflecting profound proteomic changes accompanying during the development and progression of LUAD (Fig. 2D). A subset of 1,308 proteins showed significant variation in expression levels between tumors and NATs (Fig. 2E). Specifically, 855 proteins were up-regulated while 453 proteins were down-regulated in tumors compared to NATs (Fig. 2E). GSEA brought to light the involvement of these differentially expressed tissue proteins in key biological pathways, including those of the ribosome, ECM-receptor interactions, adhesion plaque formation, amino acid biosynthesis, and pyrimidine metabolism (Fig. 2F). Extensive research suggests the tumor-associated ECM, a non-cellular component of the tumor microenvironment, interacts with cell surface receptors to promote tumor cell proliferation, invasion, metastasis, and angiogenesis [22,23]. Furthermore, focal adhesions, structures that facilitate adhesion between cells and the ECM, are integral to signaling and cell motility [24,25]. Collectively, these insights propose that exosomes released by tumors may substantially contribute to lung cancer progression, possibly by mediating intercellular communication that weakens tumor-stroma adhesion, thus endowing tumor cells with invasive and migratory properties.

### 3.3 Exosomes from LUAD cells enhance invasion and migration of parental cells

To elucidate the effect of exosomes derived from LUAD cells (LUAD-Exos) on the invasiveness and migratory abilities of the parental cells, we harvested culture supernatants from two LUAD cell lines (A549 and H1299) and conducted subsequent invasion and migration assays. The LUAD cells were co-incubated with FBS-free medium (a), the above supernatant (b), or FBS-free medium containing 10 μM GW4869 (c) (Fig. S4A). The assays indicated that exposure to supernatants significantly increased both invasion and migration capabilities of the LUAD cells in comparison to the FBS-free medium alone. In contrast, the motility of cell was markedly decreased upon treatment with GW4869, an exosome inhibitor (Fig. S4). Notably, GW4869 did not negatively affect LUAD cell viability at the applied concentration, suggesting that the observed dampening of invasive behavior in GW4869-treated cells was not due to alterations in cell proliferation or survival (Fig. S4).

**Fig. 3:**
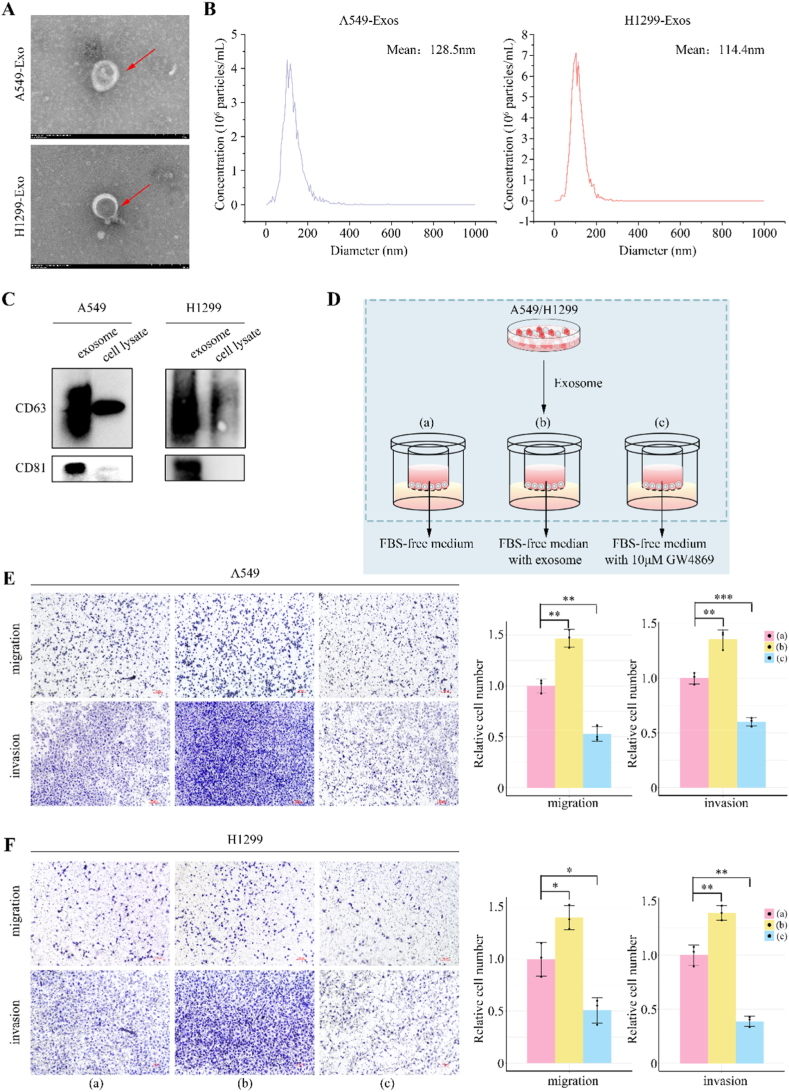
Exosomes from LUAD cells enhance invasion and migration of parental cells. (A) Supernatants from A549 and H1299 cell cultures were harvested and centrifuged to eliminate cellular debris and larger vesicles. Transmission electron microscopy (TEM) was then used to examine the morphology of the exosomes (red arrows). Scale bar: 200 nm. (B) Nanoparticle tracking analysis (NTA) was conducted to characterize the exosomes. The X-axis represented the diameters of the exosomes, and the Y-axis indicated their concentration (particles/mL) of the exosomes, and values were displayed as mean. (C) Western blot analysis was carried out to verify the presence of classic exosome markers (CD63 and CD81) in purified exosomes from A549 and H1299 cells. An equivalent amount of cell lysate was added as a negative control. (D) Experimental schematic highlighted the approach used to assess the influence of A549 and H1299 cell-derived exosomes on the invasion and migration capacities of the parent LUAD cells. (E and F) The impact of exosomes on the migratory and invasive behavior of A549 (E) and H1299 (F) cells was quantified using trans-well assays. Representative images from the assays were shown in the left panel, with the corresponding statistical evaluations in the right panel. Scale bar: 100 μm. Data are expressed as mean ± standard deviation (SD). ∗, *p* < 0.05; ∗∗, *p* < 0.01; ∗∗∗, *p* < 0.001.

Next, exosomes isolated and purified from supernatant of LUAD cells through ultracentrifugation exhibited typical morphology and size characteristic (Fig. 3A). NTA discerned circular membrane-bound vesicles with average diameters of 128.5 nm for A549-derived exosomes and 114.4 nm for H1299-derived exosomes (Fig. 3B). Western blot analysis confirmed the presence of exosome markers CD63 and CD81 within the isolated exosomes using cell lysates for comparison (Fig. 3C and S6). Subsequently, we incubated parent LUAD cells with an equal quantity of these purified exosomes to examine the effects on invasion and migration. Impressively, the isolated exosomes from the same cell lineage significantly promoted the invasiveness and migratory behavior of the parental cells, outperforming the effects seen with just the supernatant treatment (Fig. 3D–F). Taken together, our results endorse the premise that LUAD-derived exosomes inherently possess the capability to propel their parental cells towards a more invasive and high-mobility phenotype in vitro.

### 3.4 Integrated proteomic analysis of patients with LUAD

**Fig. 4:**
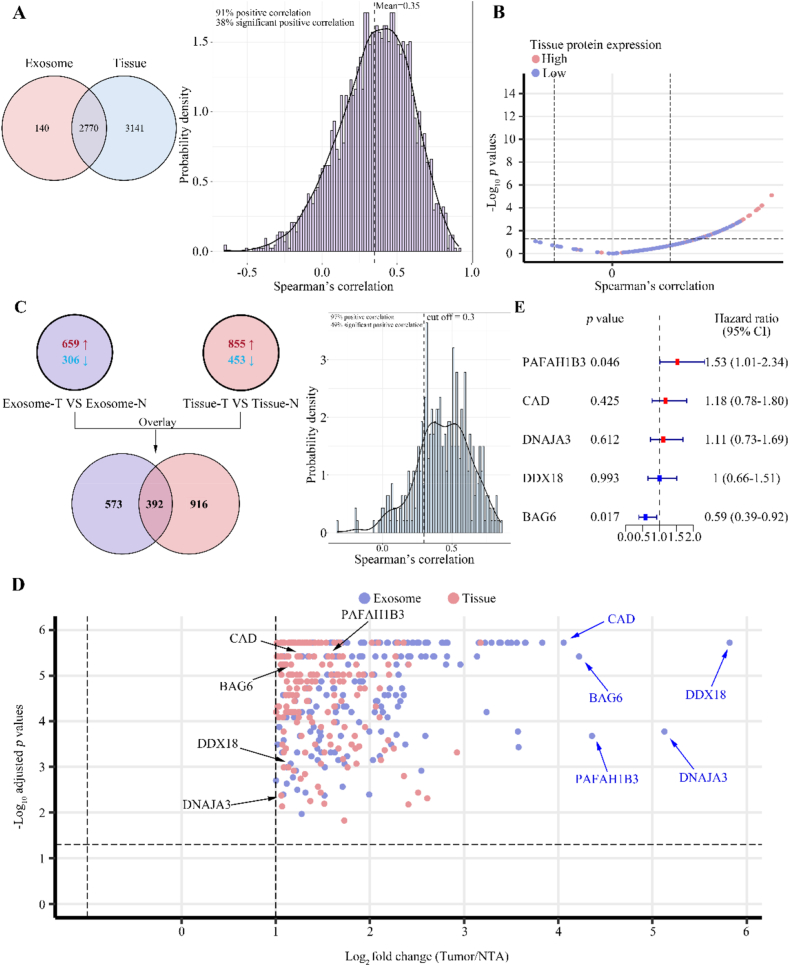
Integrated proteomic analysis of patients with LUAD. (A) Venn diagram displayed 2770 proteins with accurate tissue and exosome protein measurements (left panel). Majority of protein pairs (91 %) exhibited a positive correlation in tumor tissues and exosomes, with an average Spearman's correlation coefficient of 0.35, while only 38 % of these correlations were deem statistically significant (*p* < 0.05) (right panel). (B) Volcano plot indicated that release patterns of proteins vary according to their distinct expression patterns. (C) Proteomics identified a total of 392 differentially expressed proteins that were commonly found in both tissues and exosomes, exhibiting a positive correlation. (D) Volcano plot illustrated the fold change values (log 2 transformed) and *p*-values (-log 10 transformed) of proteins that exhibited high expression in LUAD tumors and were enriched in exosomes. (E) Effect of the expression of 5 genes (*PAFAH1B3*, *CAD*, *DNAJA3*, *DDX18* and *BAG6*) on disease-free survival (DFS) in Kaplan–Meier Plotter (KMP) databases was analyzed using a forest plot.

Exosomes, naturally occurring nanovesicles secreted by cells, encase a rich array of proteins and other constituents that mirror the cellular make-up. Consequently, we postulated a significant intersection and positive correlation in the abundance profiles of proteins between exosomes and their parent tissues. Addressing missing values, our integrated examination of proteomes derived from 20 paired tumor tissues and NATs led to the identification of 2770 proteins with reliable measures in both tissue and exosomal samples (Fig. 4A). Building on this dataset, we assessed the concordance of abundances for these 2770 proteins across tissues and exosomal specimens (TableS4). Notably, 91 % of proteins shared a positive relationship, while 38 % demonstrated a statistically significant correlation (*p* < 0.05) (Fig. 4A). The average Spearman's correlation coefficient, which measured the variability consistency between exosomal and tissue proteins, stood at 0.35. These findings affirmed the intrinsic endogenous character of exosomes and indicated a high degree of concordance between the repertoire of proteins they transport and the respective source tissue. However, a conspicuous correlation in the levels of only 38 % of these proteins between the exosomes and originating tissues points to a selective mechanism by which tumor tissues export proteins in exosomes. Further analysis of the correlations uncovered a robust consistency and substantial links for proteins with high expression in tissues and their exosomal counterparts. Nonetheless, this pattern of coherence diminished among proteins with lower tissue expression levels (Fig. 4B). These observations lend support to the notion that protein release patterns into exosomes are a reflection of cellular expression profiles, exhibiting marked fidelity particularly in the high-expression categories.

Considering LUAD tumors, we hypothesized that proteins richly transported via exosomes may serve as signaling agents promoting invasion and metastatic potential, thereby fostering an environment amenable for cancer dissemination. Confirming our proposition, we pinpointed differentially expressed proteins within both tumor tissues and exosomes, with 392 proteins showing substantial changes and positive correlations in both contexts (Fig. 4C). Among them, proteins such as DEAD-box RNA helicase 18 (DDX18), DnaJ heat shock protein family (Hsp 40) member A3 (DNAJA3), platelet activating factor acetylhydrolase 1b catalytic subunit 3 (PAFAH1B3), BAG cochaperone 6 (BAG6), and carbamoyl-phosphate synthetase 2 (CAD) not only were highly expressed in LUAD tissues but also markedly secreted into the tumor microenvironment (Fig. 4D). Clinically, disease-free survival (DFS) serves as an index to gauge patient outlook and metastatic activity. Harnessing RNA-seq data from Kaplan–Meier Plotter (KMP) databases for LUAD, we examined the effects of the expression levels of the aforementioned genes on DFS. Through Cox proportional hazard modeling, we discerned that elevated expression of PAFAH1B3 was linked to an aggravated risk for LUAD (*p* < 0.05), while augmented levels of BAG6 seemingly endowed defensive benefits (*p* < 0.05). It is worth mentioning that CAD, DNAJA3, and DDX18 did not exhibit significant ties to patient prognosis (Fig. 4E). These findings intimate an active role for PAFAH1B3 in the progression and metastasis of LUAD malignancies.

### 3.5 Exosomes from *PAFAH1B3* knockdown LUAD cells hinder cellular invasion and migration

**Fig. 5:**
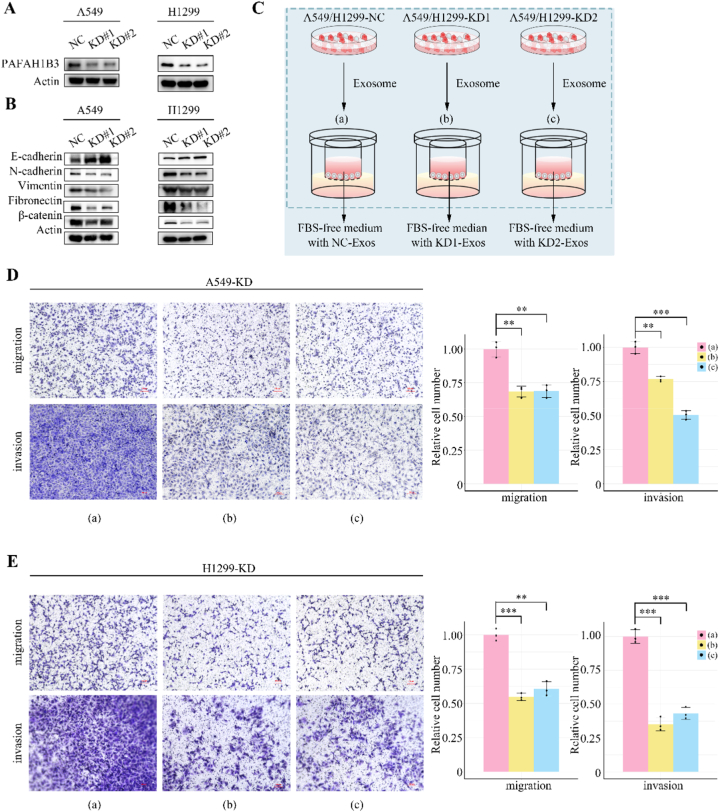
Exosomes from PAFAH1B3 knockdown LUAD cells hinder cellular invasion and migration. (A) Knockdown of PAFAH1B3 in A549 and H1299 cells were verified using Western blot. (B) Effect of PAFAH1B3 knockdown on EMT markers in LUAD cells. (C) Illustrative diagram of cell invasion and migration assays conducted to evaluate the impact of PAFAH1B3 depletion in exosomes on cellular motility. (D and E) Exosomes released by control cells (a: NC-Exos) and two types of PAFAH1B3-deficient LUAD cells (b: PAFAH1B3-KD#1-Exos; c: PAFAH1B3-KD#2-Exos) were collected and incubated with LUAD-PAFAH1B3-KD cells, respectively, to verify the effects of PAFAH1B3-deficient exosomes on the migration and invasion ability of A549 (D) and H1299 (E). Scale bar: 100 μm. Data are expressed as mean ± SD. ∗, *p* < 0.05; ∗∗, *p* < 0.01; ∗∗∗, *p* < 0.001.

As previously noted, PAFAH1B3 was markedly upregulated in LUAD tissues and predominated within exosomes, hinting at its pivotal role within the tumor microenvironment mediated by exosomal dissemination. Our current research endeavors sought to elucidate the functional significance of exosomal PAFAH1B3 in the metastatic cascade of LUAD. For this purpose, we deployed two unique guide RNAs to effectuate PAFAH1B3 suppression via the CRISPR-Cas9 system in A549 and H1299 LUAD cell lines (Fig. 5A and S6). Given the critical influence of epithelial-to-mesenchymal transition (EMT) in metastatic dynamics [[26], [27], [28]], we hypothesized that PAFAH1B3 may be implicated in the orchestration of EMT. To investigate this, we conducted Western blot analyses on an array of EMT markers (E-cadherin, N-cadherin, Vimentin, Fibronectin) as well as on the pivotal transcriptional regulator β-catenin. Post-*PAFAH1B3* depletion, an evident upsurge in E-cadherin levels was accompanied by considerable suppression of N-cadherin, Vimentin, Fibronectin, and β-catenin (Fig. 5B and S6).

To delve deeper into the influence exerted by exosomal PAFAH1B3 on metastasis, exosomes were meticulously isolated and purified from the culture supernatants of both control and *PAFAH1B3*-deficient LUAD cells. Comprehensive characterization (TEM, NTA, and Western blot) of exosomes released from control and *PAFAH1B3*-deficient LUAD cells (NC-Exos, PAFAH1B3-KD#1-Exos, PAFAH1B3-KD#2-Exos) was presented in the Supplementary Figs. 5 and 6. Subsequently, to assess the role of these exosomes in cellular migration, LUAD cells with *PAFAH1B3* knockdown were incubated with identical quantities of the three distinct exosome variants (Fig. 5C–E). According to our results, deficiency of PAFAH1B3 in LUAD exosome could significantly reverse its promoting effects on migration and invasion. In summary, these outcomes demonstrated that removing PAFAH1B3 not only curtails the inherent migration and invasion aptitudes of the cells but also undermines their capacity to augment intercellular communication via the exosomal release pathway.

### 3.6 Elevated expression of PAFAH1B3 is linked to adverse outcomes in LUAD

**Fig. 6:**
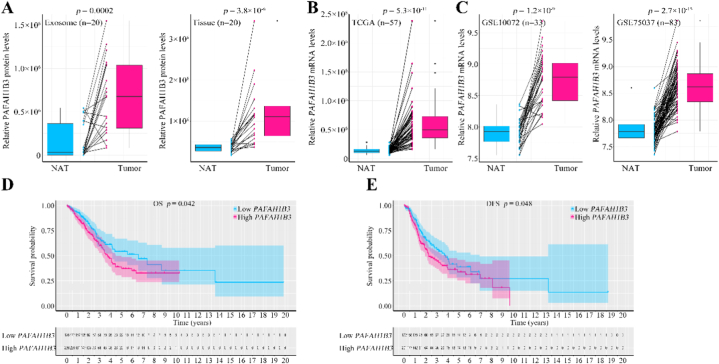
Elevated expression of PAFAH1B3 is linked to adverse outcomes in LUAD. (A) Expression of PAFAH1B3 in the exosomal proteome (left panel) and tissue proteome (right panel). (B) Analysis of The Cancer Genome Atlas Program (TCGA) dataset (n = 57) revealed upregulated mRNA expression of *PAFAH1B3* in LUAD. (C) Two Gene Expression Omnibus (GEO) databases, namely GSE10072 (n = 33) and GSE75037 (n = 83), exhibited significantly increased levels of *PAFAH1B3* mRNA in LUAD. (D and E) Overall survival (OS) and disease-free survival (DFS) curves of patients with LUAD in TCGA dataset.

Drawing from our proteomic analysis, we discerned a pronounced overexpression of PAFAH1B3 within LUAD tumors (90 %, 18/20) and detected a substantial presence of this protein in exosomes emanating from the tumor tissues (85 %, 17/20) (Fig. 6A). Pursuing a deeper insight into the role of PAFAH1B3 in LUAD, we investigated its transcriptional levels across 57 paired tumors and NATs as recorded in The Cancer Genome Atlas Program (TCGA) LUAD dataset. Notably, a substantial increase in *PAFAH1B3* mRNA expression was found in LUAD tissues compared to NATs (Fig. 6B). This overexpression was corroborated by findings in two additional LUAD cohorts from the Gene Expression Omnibus (GEO) database, specifically GSE10072 (n = 33) and GSE75037 (n = 83), which both exhibited elevated *PAFAH1B3* mRNA levels (Fig. 6C). Furthermore, prognostic implications were explored through the TCGA database, which uncovered a correlation between higher *PAFAH1B3* expression and reduced OS as well as disease-free survival (DFS) in patients with LUAD (Fig. 6D–E).

In conclusion, our data highlighted PAFAH1B3 as a potential prognostic biomarker in LUAD, pointing to its dual signature of high expression in tumor tissues and pronounced exosomal release as a harbinger of poor patient prognosis.

## 4 Discussion

Previous research on exosome proteomics in LUAD has mainly concentrated on utilizing liquid biopsies to facilitate early diagnosis and predict metastasis [[14], [15], [16], [17]]. However, the direct study of exosomes originating from LUAD tissues remain uncharted. Current omics approaches in lung cancer largely target small molecular entities, such as long non-coding RNAs, metabolites, and circular RNAs [[29], [30], [31]], leaving a gap in our proteomic understanding of LUAD and its exosomes. This proteomic perspective could potentially unravel the complex dynamics between oncogenic and autocrine proteins within the tumor microenvironment. By dissecting the proteome of exosomes from LUAD compared to NATs, we have pinpointed cancer pathways linked to LUAD-associated exosomes. Significantly, several lung-tissue-specific exosome proteins registered elevated expressions in tumors relative to NATs, potentially driving worse outcomes through pathways like the proteasome, ECM-receptor interactions, ribosomes, adhesion plaque, and lysosome. Our proteomic scrutiny of exosomes from Chinese patients with LUAD has enriched the collective insight into the exosome proteome, thereby deepening our comprehension of the disease.

While existing literature has reported relationships between tumor proteins and exosomal proteins, such studies have predominantly been confined to in vitro experiments using tumor cells and their secreted exosomes [32]. Consequently, these findings may not truly capture the intricate correlations present within lung cancer patients, often omitting a quantified assessment of these relationships [32]. To overcome these limitations, we collected tumor specimens and their corresponding NATs from the same LUAD cohort for a more comprehensive analysis. Each tissue specimen was partitioned into two segments, one allocated for examining the tissue proteome and the other for scrutinizing the exosomal proteome. Remarkably, out of the 2,770 tumor-exosomal protein pairs analyzed, a positive correlation was observed in 91 % of cases, yet only 38 % demonstrated statistically significant correlations. Additionally, we discovered diverse patterns of correlation between tissue proteins of varying expression levels and the corresponding abundance in exosomal proteins. Such findings accentuated the imperative for further exploration into the selective release of proteins by tumor cells and the possible ramifications this holds for tumor progression.

Platelet activating factor acetylhydrolase 1b catalytic subunit 3 (PAFAH1B3) has been implicated as a dysregulated metabolic gene associated with cancer processes including cell proliferation, apoptosis, and metastasis across various cancer types [[33], [34], [35], [36]]. Expression of PAFAH1B3 was crucial in hypopharyngeal non-squamous cell carcinoma for modulating cell proliferation, migration, invasion, apoptosis, and cell cycle disruption [37]. It may also play a role in liver cancer progression through effects on cell cycle control, metabolism, spliceosome functions, and RNA transport [35]. Moreover, PAFAH1B3 was linked to the metastasis of thyroid papillary cancer via the EMT pathway [38]. Despite these associations, the role of PAFAH1B3 in LUAD metastasis through the exosomal route has remained uncharted territory. In our study, we deployed exosomes from *PAFAH1B3*-KD LUAD cells to investigate how exosomal PAFAH1B3 influences the motility of LUAD cells. Co-culturing tumor cells with these modified exosomes led to markedly diminished migratory and invasive potentials. Moreover, PAFAH1B3 was significantly overrepresented not just in tumor tissues and their released exosomes but also displayed abnormally elevated mRNA levels in the TCGA LUAD database and two GEO cohorts, positioning it as a strong indicator of dismal patient prognosis.

Tumor metastasis is a complex and multi-stage process that primarily encompasses the phases of invasion, dissemination through circulation, and eventual colonization at distant sites [39]. During the invasion stage, tumor cells present within the original tumor site amplify their invasiveness through undergoing the EMT process. This transition enables them to invade surrounding tissues, migrate towards nearby blood or lymphatic vessels, breached the blood vessels to enter the circulatory system, and subsequently transformed into circulating tumor cells [40]. Owing to tumor heterogeneity and the intratumoral heterogeneity among different cells within the same tumor, differential gene expression manifests across various cell subpopulations. We hypothesized that tumor cells might acquire certain phenotypes from other tumor cells through exosomal secretion, thereby initiating metastatic cascade reaction that led to the departure of tumor cells from the primary tumor site. In our study, the exosomal protein PAFAH1B3 was served to augment the motility of cells within the tumor cell mass that exhibit low expression of PAFAH1B3. Consequently, PAFAH1B3 has the potential to act as a therapeutic target for intervention in the incipient phases of tumor metastasis.

## 5 Conclusions

Our research constitutes the inaugural creation of a comprehensive proteomic profile encompassing both tumor tissue and secreted exosomes from Chinese patients with LUAD. Through rigorous correlation analysis of the proteome within the tissues and their associated exosomes, we have revealed indications of selective protein encapsulation into exosomes. Most notably, with corroborating data analysis and in vitro validation, PAFAH1B3 is identified as not merely overexpressed but also stands as a promising candidate for a potent prognostic biomarker and therapeutic target in lung cancer.

## CRediT authorship contribution statement

**Congcong Wang:** Writing – original draft, Methodology, Conceptualization. **Ling Xiao:** Software. **Ling Gao:** Methodology. **Jia Wu:** Methodology. **Siliang Wang:** Methodology. **Miao-Miao Zheng:** Methodology. **Chen-Tai Qin:** Methodology. **Xian-ge Huang:** Methodology. **Lei Zhou:** Methodology. **Wei-jie Xu:** Methodology. **He-gen Li:** Methodology. **Wen-Lian Chen:** Methodology. **Li-hua Zhu:** Conceptualization. **Xing Jin:** Writing – review & editing, Data curation, Conceptualization.

## Ethics committee approval and patient consent

The research was conducted in accordance with the Declaration of Helsinki and approved by the Ethics Committee of Affiliated Tumor Hospital of Nantong University (ATHNU) (2022-A06, date of approval: July 12, 2022). Written informed consent was obtained from the patients.

## Data availability statement

Data included in article/supp. material/referenced in article.

## Declaration of AI and AI-assisted technologies in the writing process

The authors did not use AI or AI-assisted technologies during the writing process.

## Funding

This work was supported by the National Key R&D Program of China (2022YFC3500200, 2022YFC3500202), 10.13039/501100001809National Natural Science Foundation of China (31970708, 82002953, 32170778, 82004177, 82072567, 82304780, 82205214), Shanghai Frontier Research Base of Disease and Syndrome Biology of Inflammatory cancer transformation (2021KJ03-12), 10.13039/501100013076National Scientific and Technological Major Special Project of China (2019ZX09201004-002-013), Tracking Program for Eastern Scholar at Shanghai Institutions of Higher Learning, Shanghai High-level Talent Leadership Program of Traditional Chinese Medicine [ZY(2021-2023)-0403], Scientific Research Project of Industry Development Center of 10.13039/501100010876Shanghai University of Traditional Chinese Medicine (602076D), the grant from Nantong Health Commission (MA2021024), Shanghai “Science and Technology Innovation Action Plan” Medical Innovation Research Project-10.13039/501100016983Shanghai Clinical Research Center of Traditional Chinese Medicine Oncology (21MC1930500), Shanghai 13th Five-Year Plan Key Specialty of Traditional Chinese Medicine Oncology (shslczdzk03701), Three-year Action Plan for Shanghai TCM Development and Inheritance Program [ZY(2021-2023)-0401], and Health Commission of Pudong New Area Health and Family Planning Scientific Research Project (PW2019E-1).

## Declaration of competing interest

The authors declare that they have no known competing financial interests or personal relationships that could have appeared to influence the work reported in this paper.
