# Supplementary material for: Comparative proteomic analysis between tumor tissues and intratumoral exosomes from lung adenocarcinoma patients identifies PAFAH1B3 as an exosomal protein key for initiating metastasis in lung adenocarcinoma
Source: Heliyon. 2024 Oct 28;10(21):e39859. doi: 10.1016/j.heliyon.2024.e39859 (PMC11567031; doi:10.1016/j.heliyon.2024.e39859)
Supplement: Multimedia component 1 [file mmc1.pdf]

Figure S1

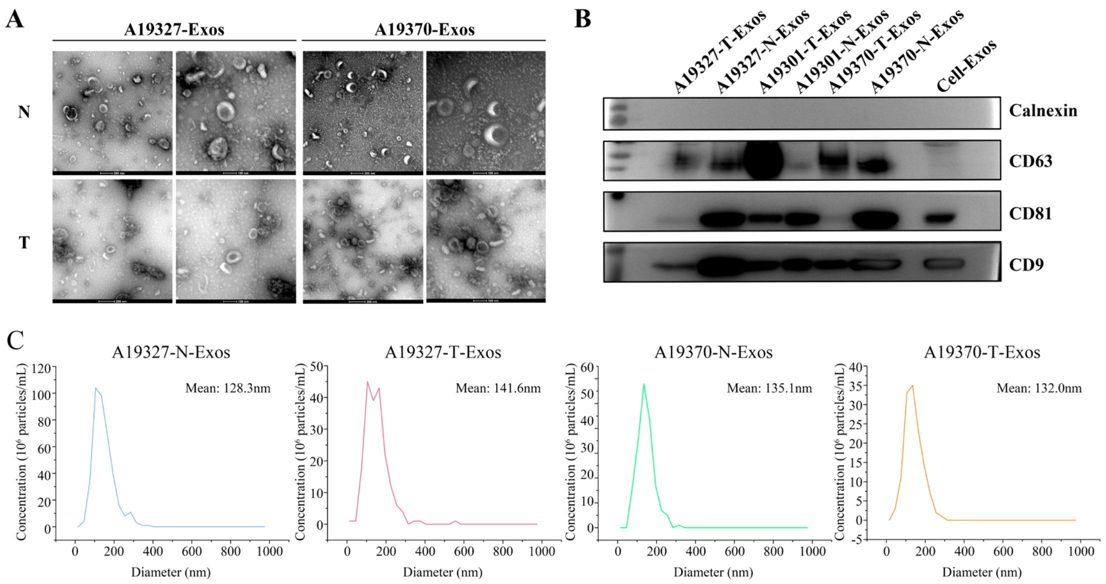

(A) Exemplary TEM images illustrating exosomes isolated from the tumor tissues and NATs of patients with LUAD. Scale bar: left, 200 nm; right, 100 nm.

(B) Western blot analysis was performed to identify exosomes from tissues employing CD63, CD81 and CD9 as positive exosomal markers, while Calnexin served as a negative marker.

(C) Quantitative assessment through NTA was conducted on tissue-derived exosomes of individuals diagnosed with LUAD.

Figure S2

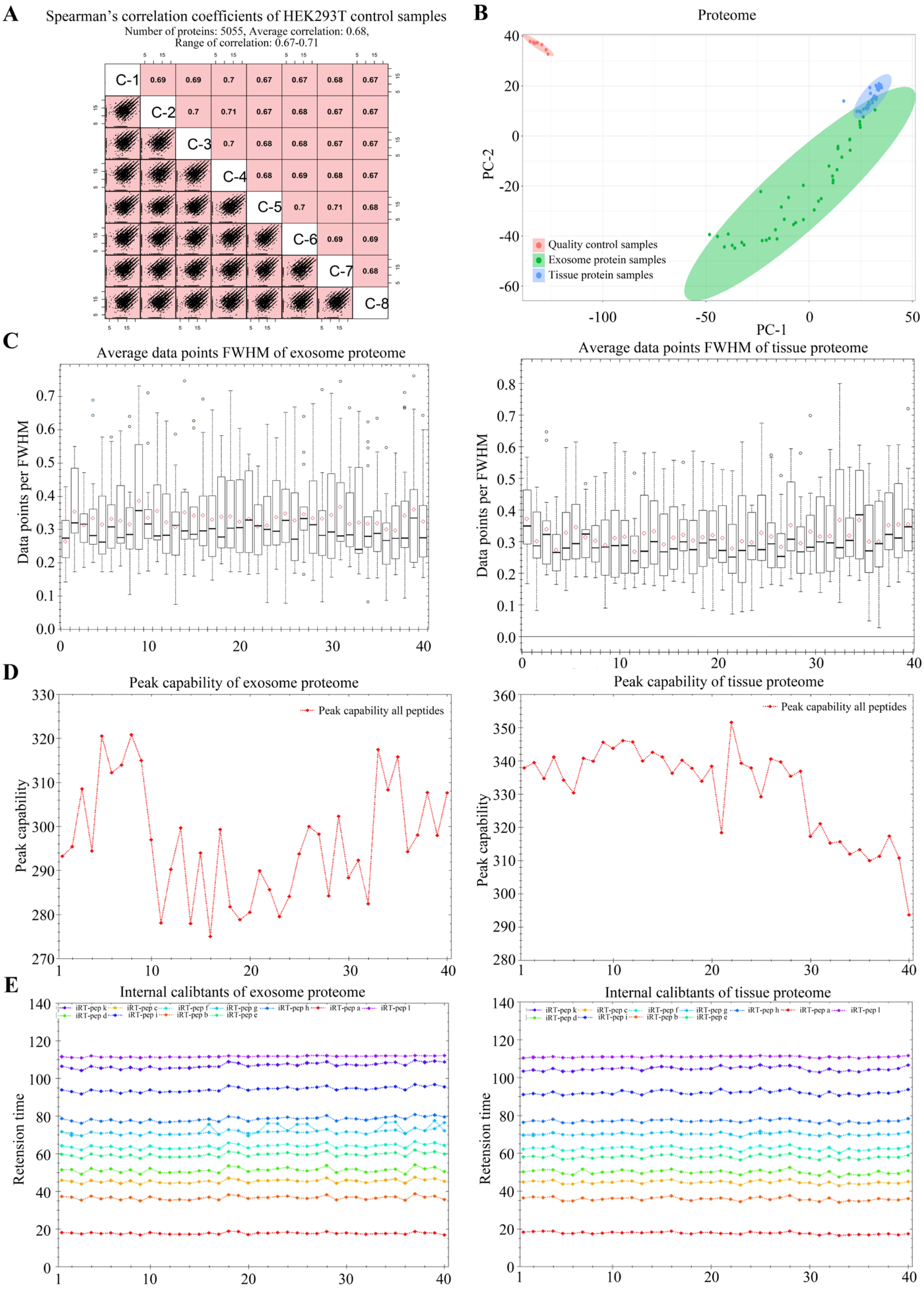

(A) Correlation analysis on eight HEK293T cell samples was conducted for longitudinal mass spectrometry quality control. The bottom-left section features scatter plots for pairwise sample comparisons, while the top-right section shows the Spearman's correlation coefficient values for each corresponding sample pair.

(B) Principal component analysis (PCA) was performed on all protein samples,

including QC samples, tissue samples and exosome samples. The concentrated distribution of QC samples indicated that the measurement system was consistently reliable across the analyses.

(C) FWHM of proteome samples. (Left panel: exosomes; right panel: tissues)

(D) Peak capacity of all peptides in the proteome. (Left panel: exosomes; right panel: tissues)

(E) Internal calibrants for the proteomic data. (Left panel: exosomes; right panel: tissues)

Figure S3

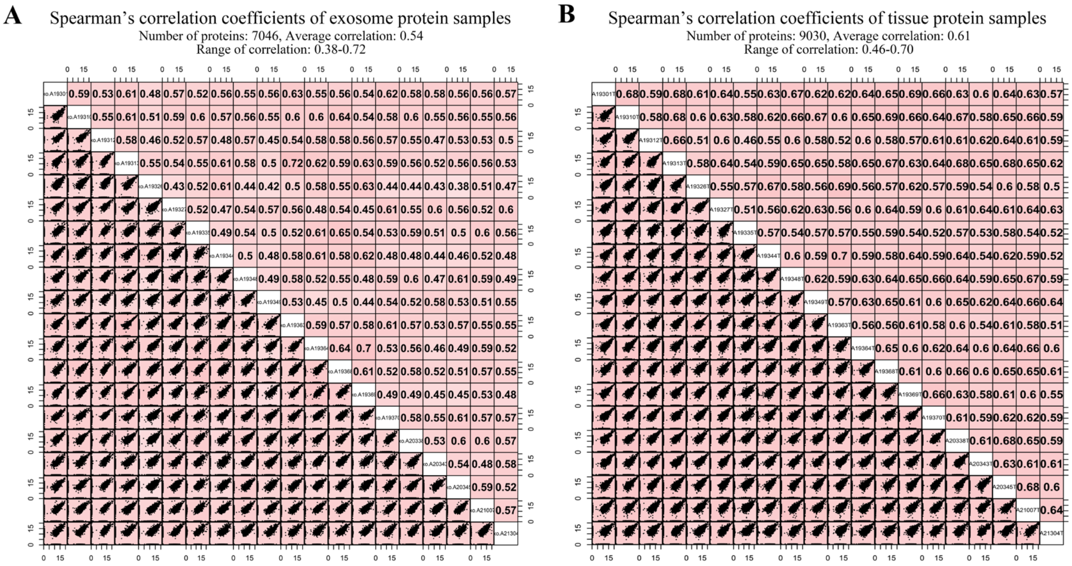

(A) Scatter plot and Spearman’s correlation coefficient analysis of exosome proteome profiles in tumors. The x and y axes indicate the strength of the Log2-transforming protein in each pair of comparisons.

(B) Scatter plot and Spearman’s correlation coefficient based on proteome analysis of tumors.

Figure S4

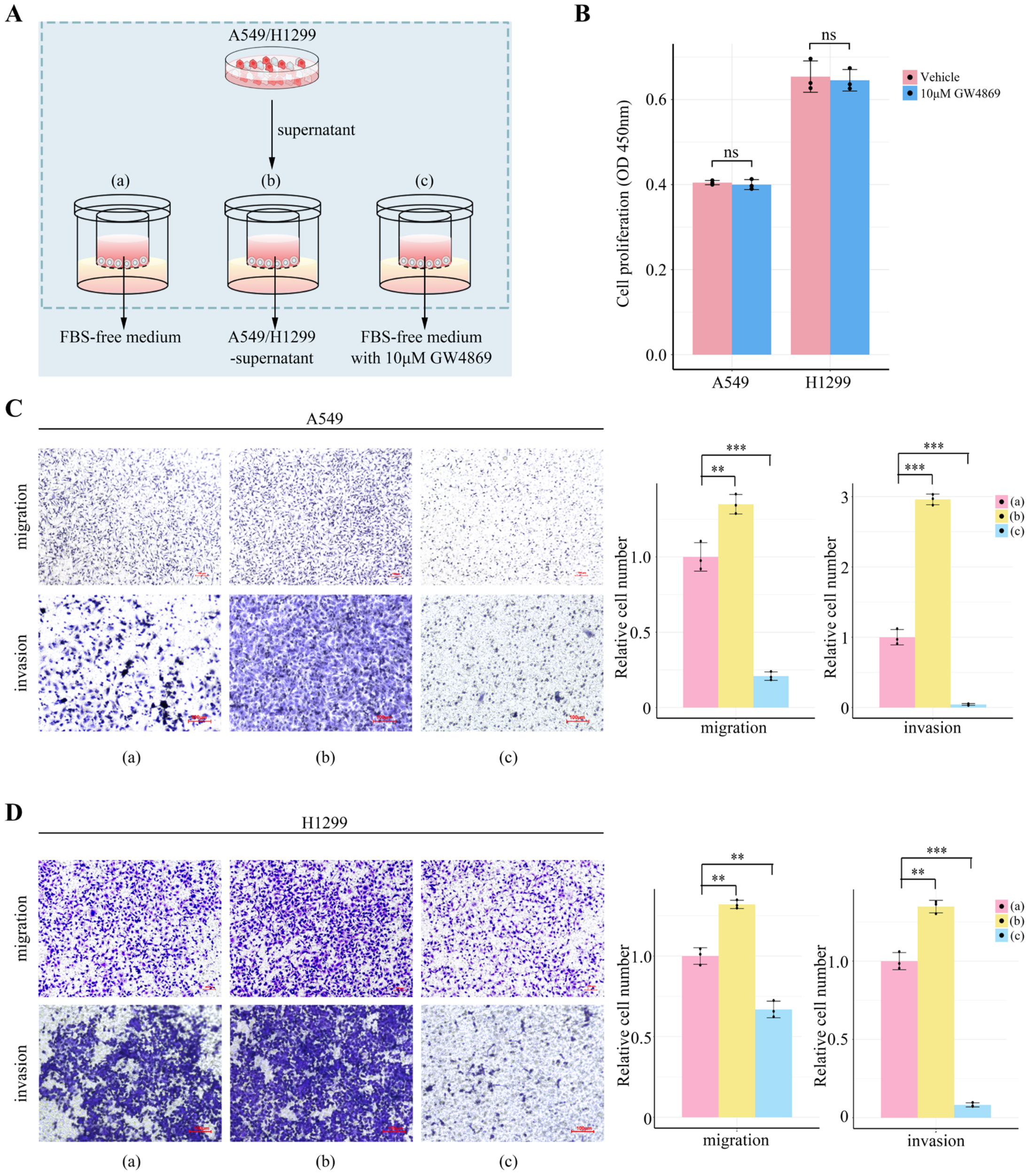

(A) A schematic overview showcasing the effects of culture supernatant from A549 and H1299 cell lines on invasion and migration potentials of parental LUAD cells.

(B) Evaluation of LUAD cell proliferation response to GW4869 treatment (10μM in FBS-free medium) over a period of 24 hours. Data were shown as mean ± standard deviation (SD), n=3. ns, non-significant.

(C and D) Effects of A549 and H1299 cell culture supernatants on the migration and invasion abilities of their parent cells. Left panel: representative images of the migration and invasion assays; right panel: statistical analysis of the migration and invasion assays. Scale bar: 100 μm. Data were presented as mean ± SD. *, $p < 0.05$; **, $p < 0.01$; ***, $p < 0.001$.

Figure S5

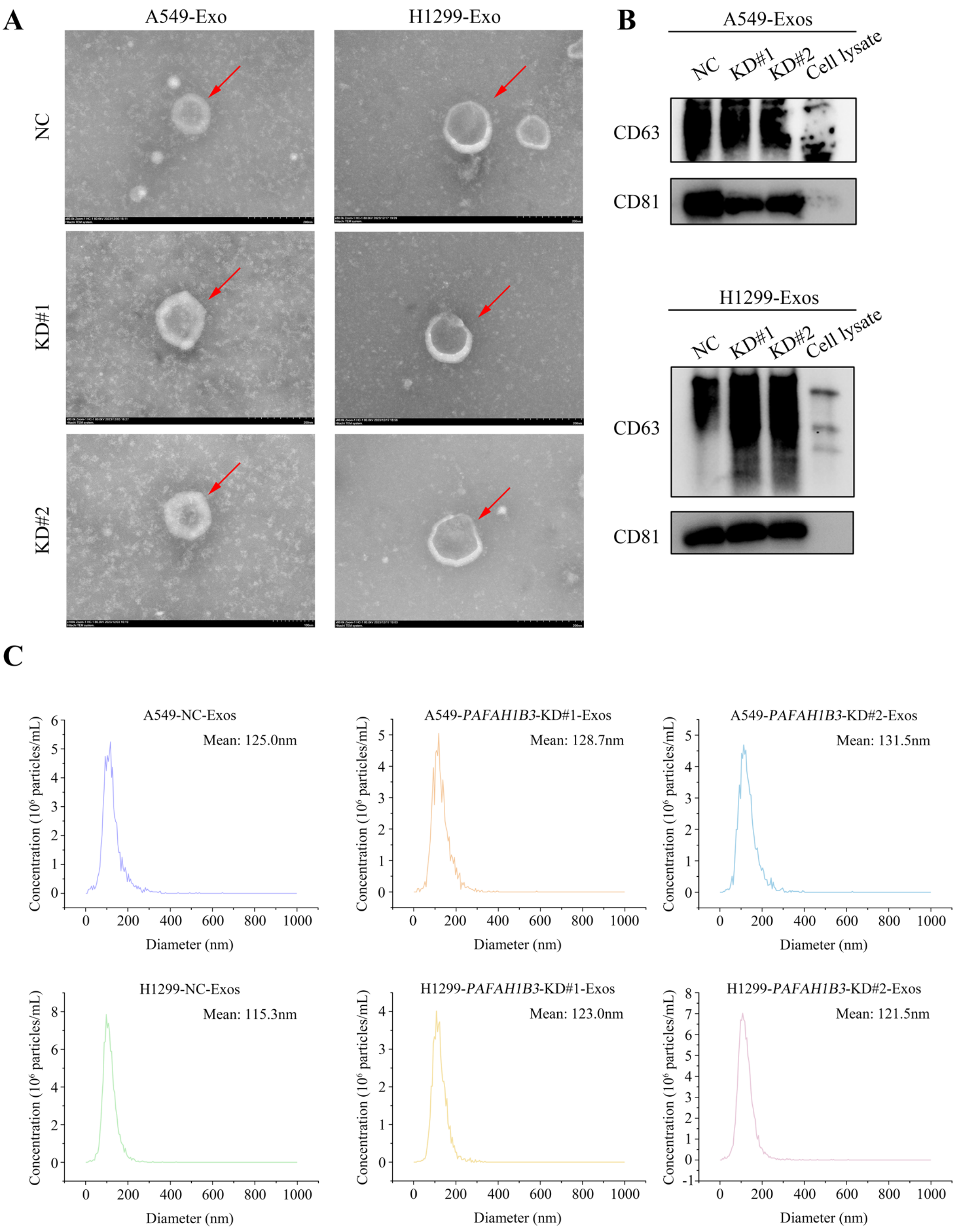

Comprehensive characterization of exosomes released by control or *PAFAH1B3*-deficient LUAD cells (NC-Exo, *PAFAH1B3*-KD#1-Exo, *PAFAH1B3*-KD#2-Exo) was conducted, including TEM (A), western blot (B) and NTA (C) identification.

Figure S6

Figure 3C

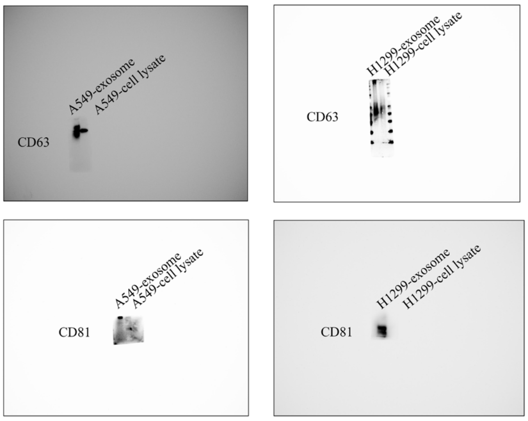

Figure 5A

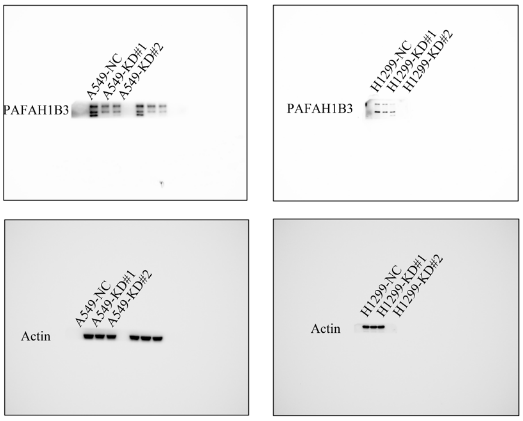

Figure 5B

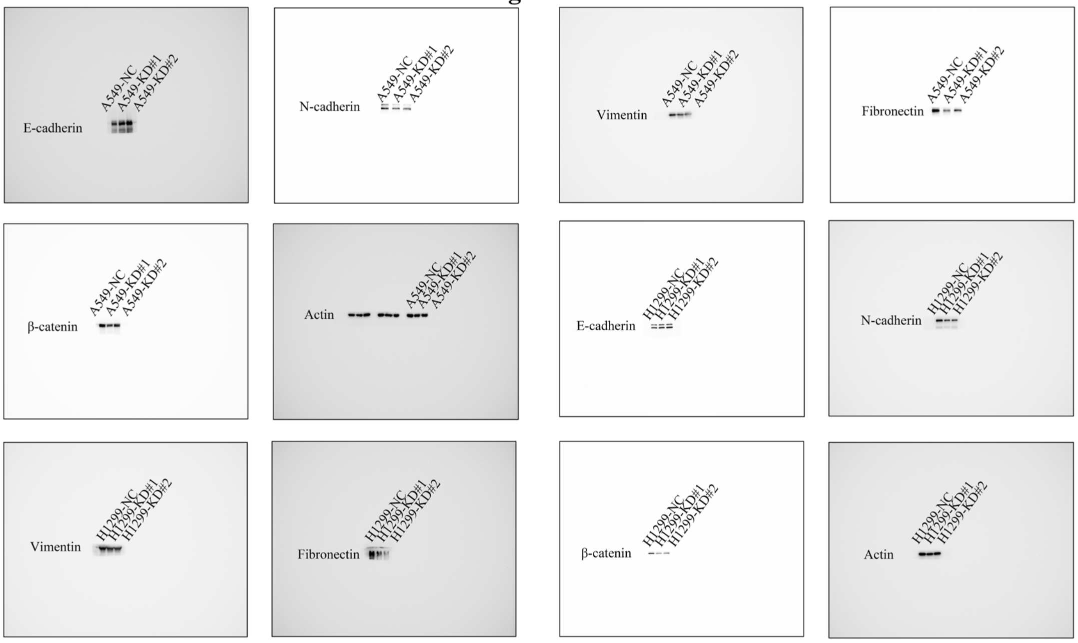

Figure S1B

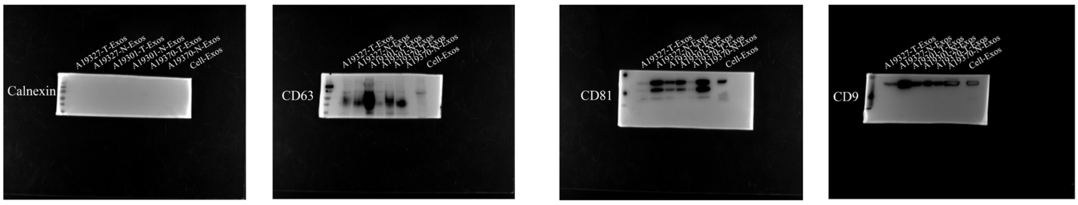

Figure S5B

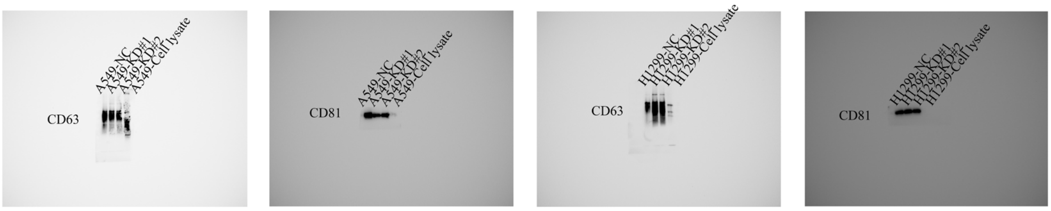

Uncropped and unadjusted images of figures (3C/ 5A / 5B/ S1B/ S5B).
