# Supplementary material for: Comparative proteomic analysis between tumor tissues and intratumoral exosomes from lung adenocarcinoma patients identifies PAFAH1B3 as an exosomal protein key for initiating metastasis in lung adenocarcinoma
Source: Heliyon. 2024 Oct 28;10(21):e39859. doi: 10.1016/j.heliyon.2024.e39859 (PMC11567031; doi:10.1016/j.heliyon.2024.e39859)
Supplement: Multimedia component 4 [file mmc4.docx]

**Table S3. Basic characteristics of patients with LUAD**

| Characteristic | LUAD (n = 20) |
| --- | --- |
| Age, years |  |
| Median (range) | 62 (46-79) |
| ≥ 60 years, no. (%) | 13 (65) |
| < 60 years, no. (%) | 7 (35) |
| Sex, no. (%) |  |
| Male | 10 (50) |
| Female | 10 (50) |
| Alcohol consumption (habitual), no. (%) |  |
| Never | 20 (100) |
| Previous | 0 (0) |
| Smoking status, no. (%) |  |
| Never smoker | 17 (85) |
| Ex-smoker | 3 (15) |
| Hypertension, no. (%) |  |
| Yes | 8 (40) |
| No | 12 (60) |
| Diabetes, no. (%) |  |
| Yes | 1 (5) |
| No | 19 (95) |
| Histological type, no. (%) |  |
| Adenocarcinoma | 20 (100) |
| Other | 0 (0) |
| Treatment protocol, no. (%) |  |
| Surgery alone | 8 (40) |
| Surgery plus adjuvant chemotherapy | 12 (60) |
| Clinical T stage, no. (%) |  |
| T1 | 4 (20) |
| T2 | 12 (60) |
| T3 | 4 (20) |
| Clinical N stage, no. (%) |  |
| N0 | 9 (45) |
| N1 | 4 (20) |
| N2 | 6 (30) |
| N3 | 1 (5) |
| Clinical stage group, no. (%) |  |
| I | 6 (30) |
| II | 7 (35) |
| III | 7 (35) |
